# The American Medical Association

**Published:** 1887-07

**Authors:** 


### THE AMERICAN MEDICAL ASSOCIATION.

The sessions of the American Medical Association, at Chicago, on the 7th, 8th, 9th and 10th of June, were attended by 1,080 delegates, with a large number of permanent members and visitors, reaching in all, perhaps, 1,500 attendants. They came from every section of the country, and Georgia was represented by Drs. Whitehead, Deckel, Hopkins, Cortelyou, Bailey, Gray, Westmoreland, and Gaston. In view of the fact that Dr. Whitehead, the president-elect of the Georgia Medical Association, was selected as the member from this State, for the nominating committee, all may rest assured that the interests of the profession were properly represented in that body.

The annual address of the president, Dr. E. H. Gregory, of Saint Louis, on "Cell Antagonism," was such a departure from the usual routine as is worthy of imitation by future presiding officers. He gave a fitting notice of the death of Dr. W. O. Baldwin, an ex-president of the Association, since the last meeting. The forthcoming meeting of the International Medical Congress, in the City of Washington, received his attention in the declaration that a hearty American welcome awaits those with whom we have heretore been united in the cause of science.

On motion of Dr. William Brodie, of Detroit, an amendment to the by-laws, providing for a section in Dermatology and Venereal Diseases, was taken from the table, and, with an amendment, proposed by Dr. G. H. Rohe, of Baltimore, substituting the word "Syphilography" for venereal diseases, was adopted.

Dr. J. McF. Gaston, of Atlanta, Ga., as chairman of the committee to Memorialize Congress with reference to Inoculation for Yellow Fever, presented a verbal report, which was referred to the section on State Medicine for further consideration.

On Wednesday, June 8—second day—Dr. J. M. Toner, of Washington City, chairman of the board of trustees for the publication of the journal of the Association, reported that the general success of the journal had been more gratifying, during the year, than could have been expected.

Dr. N. S. Davis, of Chicago, chairman of the special committee on Changes in the plan of organization and by-laws of the Association, reported in favor of a general committee of two members from State and Territorial medical societies represented by delegates, and from the Army, Navy and Marine Hospital service, chosen by the members at the annual meeting. This general committee shall nominate, on the third day of each annual meeting, all the officers (none of whom shall be members of their own body), the judicial council, the board of trustees for the Journal, etc. It was recommended that the by-laws be so altered that the chairmen of sections shall read their addresses before the several sections, and not occupy more than forty minutes. It was also provided that three members of the profession shall be elected by the Association to deliver addresses in the general sessions of the Association at the next annual meeting, and that each shall be limited to one hour. After sundry propositions and irregularities it was finally agreed that the constitutional amendments lie over a year, and that the proposed changes in the by laws be adopted and go into effect forthwith.

While it may be claimed that "all's well that ends well," the proceedings in this matter in sanctioning a violation of the constitutional provision that all proposed changes of the organic law shall lie over for twelve months, must be regarded as a lamentable departure from the dignity and order which ought to characterize the action of this Association.

The following proposition by the American Pharmaceutical Association met a hearty approval:

"*Resolved*, That this Association solicits the aid and co-operation of the American Medical Association in promoting the prescribing of officinal medicines only, or such preparations as have published formulas, in preference to others."

The address of the chairman of the section in the Practice of Medicine by Dr. John S. Lynch, of Baltimore, took issue with Koch's theory, and he denied that the bacillus is the sole or frequent cause of phthisis.

On Thursday, June 8—third day—Dr. William Brodie, of Detroit, chairman of the committee on nominations, made the following report:

President—A Y. P. Garnet, of Washington.

First Vice-President—Duncan Eve, Nashville, Tenn.

Second Vice-President—Darwin Calvin, Clyde, N. Y

Third Vice-President Charles J. O. Hagan, North Carolina.

Fourth Vice-President—A. Stedman, Colorado.

Librarian—C. H. A. Kleinschmidt, Washington.

Treasurer—R. J. Dunglison, Philadelphia.

Permanent Secretary—W. B. Atkinson, Philadelphia.

Assistant Secretary—Joseph Ransahoff, Cincinnati.

Trustees of the Journal—Leartus Connor, Detroit; E. O. Shakespeare, Philadelphia; W. T. Briggs, Tennessee

Judicial Council—J. H. Murphy, St. Paul; J. M. Toner, Washington; J. K. Bartlett, Milwaukee; A. B. Sloane, Missouri; X. C Scott, Cleveland; A. W. McLuer, Iowa; D. W. Stormont, Kansas; J. H. Hibberd, Indiana.

Committee on Necrology—J. M. Toner, Chairman.

Committee on State Medicine—R. G. Jennings, Chairman.

The next annual meeting is to be held at Cincinnati on the second Tuesday in May, 1888. Chairman of Committee of Arrangements, Dr. W. W. Dawson, of Cincinnati, with power to fill the Committee by appointment.

Officers elected by the Sections:

Surgery—Donald McLean, Ann Arbor, chairman; B. A. Watson, Jersey City, N. J, secretary.

Medicine—A. B. Palmer, Ann Arbor, chairman; N. S. Davis, jr, Chicago, secretary.

State Medicine—H. B. Baker, Lansing, Mich., chairman; S. T. Armstrong, Tennessee, secretary.

Children—F. E. Waxham, Chicago, chairman; W. B. Lawrence, Batesville, Ark., secretary.

Dental and Oral Surgery—J. Tafft, Cincinnati, chairman; E. S. Talbot, Chicago, secretary.

Ophthalmology and Otology—F. E. Hotz, Chicago, chairman ; H. H. Jackson, Philadelphia, secretary.

Dermatology and Syphilography—L. Duncan Bulkley, New York, chairman ; T. Fayette Dunlap, secretary.

Medical Jurisprudence—E. M. Reid, Baltimore, chairman ; Dr. Belt, Boston, secretary.

The report of the committee on Monument to Benjamin Rush, in the absence of Dr. A. L. Gihon, chairman, was read by Dr. G. H. Rohe, showing that $389 had been received.

Dr. John Morris, of Baltimore, in the absence of Dr. J. M. Keller, of Hot Springs, Ark., read the report of the special committee on Cremation, closing with the resolution : "that it is the judgment of this Association that burial of persons dying of zymotic diseases should be placed by law under the control of health authorities."

The following resolution was presented by Dr. J. McF. Gaston, of Atlanta, Ga., and was opposed by Dr. G. H. Rohe, of Baltimore, but was adopted :

"*Resolved*, That it is the sense of this Association that it is desirable that two other members of the medical profession be associated with the Committee on Inoculation of Yellow Fever already appointed, and that a committee of three be appointed to communicate this action to President Cleveland."

The address of the section on Obstetrics and Diseases of Children was delivered by the chairman, Dr. F. M. Johnson, of Kansas City, Mo., indorsing Alexander's operation, and claiming advantages for the modified Cæsarian section over other procedures.

The address of Dr. G. H. Rohe, of Baltimore, chairman of the section on State Medicine, among other important hygienic considerations, adverted to yellow fever inoculation now under official investigation by the Government.

On Friday, June 10—fourth day—soon after the opening of the meeting, and in advance of the prescribed order for miscellaneous business, Dr. J. B. Hamilton, of Washington City, offered a preamble and resolution to this effect : That Congress be requested to furnish copies of the Report to be published by Dr. G. M. Sternberg, U. S. A., relating to his researches into the causes of yellow fever in Mexico and Brazil, and that the action of the previous day in regard to this matter be rescinded.

Dr. J. McF. Gaston, of Atlanta, Ga., claimed that this was out of order, as in a full meeting of the Association, on the previous day, the resolution proposed by him for the appointment of two additional commissioners had been sustained by a large majority against a motion to lay on the table, and against a motion to reconsider after its passage.

Dr. Duncan Eve, of Nashville, Tenn., relied upon the ruling of the president that it could not be taken up until the regular order for miscellaneous business, but not having so determined the matter was open for discussion.

Dr. Gaston then moved, and was seconded by Dr. George Grissom, of Raleigh, N. C., that the vote be taken separately upon the preamble relating to the work of Dr. Sternberg, and the resolution rescinding the previous action.

The previous question being called and sustained, the vote was taken upon the whole, resulting in its adoption. Thus a large majority of the entire body, in a full meeting on the previous day, passed the recommendation for the appointment of additional commissioners for the investigation of yellow fever inoculation in Mexico and Brazil, which is rescinded by a comparatively small number of the members present on the morning of the last day, in an irregular manner, and out of time for such business !

The address of the chairman of the section on Dental and Oral Surgery was delivered by Dr. J. S. Marshall, of Chicago.

Dr. J. M. Quimby, of Jersey City, N. J., read the address of the chairman of the section of Medical Jurisprudence, giving an outline of the history of medical jurisprudence from the early periods of Hebrew, Greek and Roman antiquity.

The Medical Congress asked for an appropriation of money. After stirring speeches, in which were set forth the pressing and urgent financial needs of the Congress, one thousand dollars were appropriated, and it was earnestly hoped that every member would make immediate and liberal contributions, besides the amount required for membership.

The Preservation of Fœtal and Infantile Life was then considered, and he suggested that a special committee be appointed by the Association to report at the next annual meeting upon the criminality of Fœticide and such measures as may be commended for legislative action for its prevention and punishment. Attention was directed to the duties exercised commonly by coroners and the means of improving medical police in regard to Immigration, with special reference to the detection and prohibition of chronic invalids and insane persons.

The address closed with a special reference to the medical jurisprudence of Drunkenness.

Dr. J. M. Toner, of Washington, presented the report of the committee on Neurology.

Dr. N. S. Davis, of Chicago, reported from the standing committee on Meteorological Conditions and their relations to the prevalence of disease ; also concerning the collective investigation of disease in co-operation with the committee of the British Medical Association.

On motion of Dr. N. S. Davis, of Chicago, the following resolutions were adopted :

"First. That regular graduates of dental and oral schools and colleges which required of their students a standard of preliminary or general education to the term of professional study be recognized as members of the regular profession of medicine and eligible to membership in this Association on the same conditions and subject to the same regulations as other members.

"Second. That the Committee of Arrangements are hereafter directed, at each annual meeting of this Association, to so arrange the programme regarding entertainments and receptions that the evening of the third day be reserved for the regular annual dinner, under such regulations that the members may dine with or without wine, according to choice, and pay only for what they elect to have furnished, and that this be the entire cost of the dinner, leaving no part to be paid either by the local profession or the treasurer of the Association."

It may be observed that the elegant entertainments and receptions given to the members of the Association during their stay in Chicago were for the most part without wine or other spirituous liquors and all were greatly enjoyed by the visitors. In view of this, and the good results of banquets elsewhere without wine, it will strike many with surprise that the above action was taken by the Association.

The work of the sections was more than usually interesting, by the number and the character of the contributions; but our space does not admit of even a glance at the many instructive papers presented, and we content ourselves with simply stating that our fellow townsman Dr. J. McF. Gaston read an elaborate paper before the section on Surgery, entitled "The Pathology, Diagnosis and Treatment of Perforation of the Appendix Vermiformis." We trust that it may be in our power to present a synopsis of this paper in our next number, and to give extended notices of some of the other valuable papers read before the sections.

